# Supplementary material for: Lymphocyte subsets in Atlantic cod (Gadus morhua) interrogated by single-cell sequencing
Source: Commun Biol. 2022 Jul 11;5:689. doi: 10.1038/s42003-022-03645-w (PMC9276791; doi:10.1038/s42003-022-03645-w)
Supplement: Supplementary file 2 — Supplementary Information [file 42003_2022_3645_MOESM2_ESM.pdf]

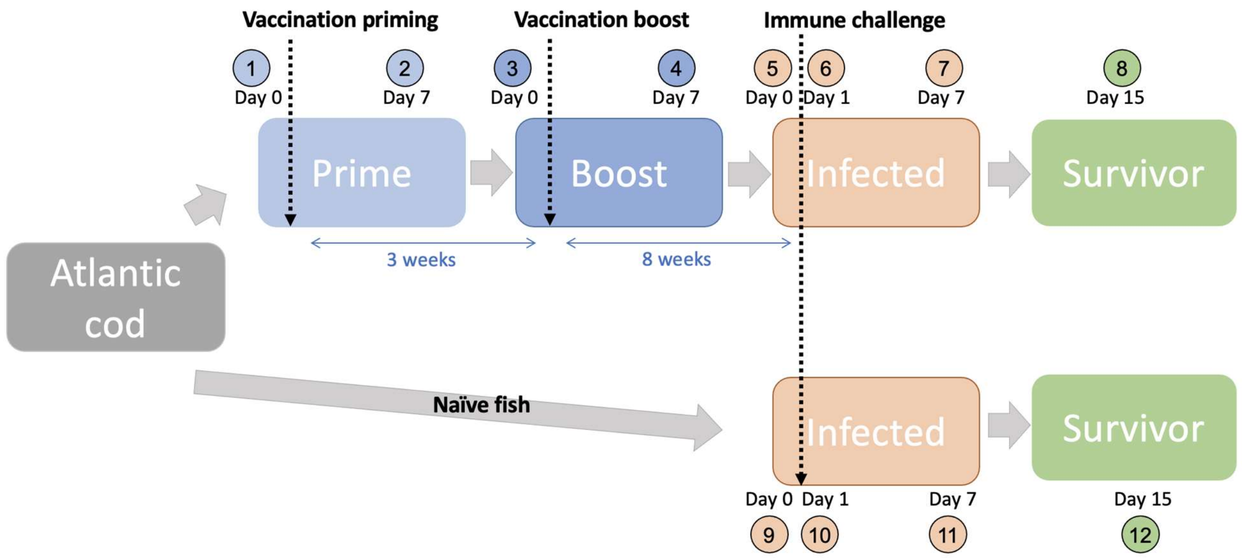

Supplementary Figure 1.

Schematic showing sampling timeline.
Atlantic cod were split into two groups: vaccinated and naïve fish. Following priming and boost immunisations, both groups were challenged by the pathogen *Vibrio anguillarum* and sampled on days 1, 7 and 15 after challenge. Each sampling point, 1-12, represents 2-3 fish. 34 Atlantic cod were sampled in total.

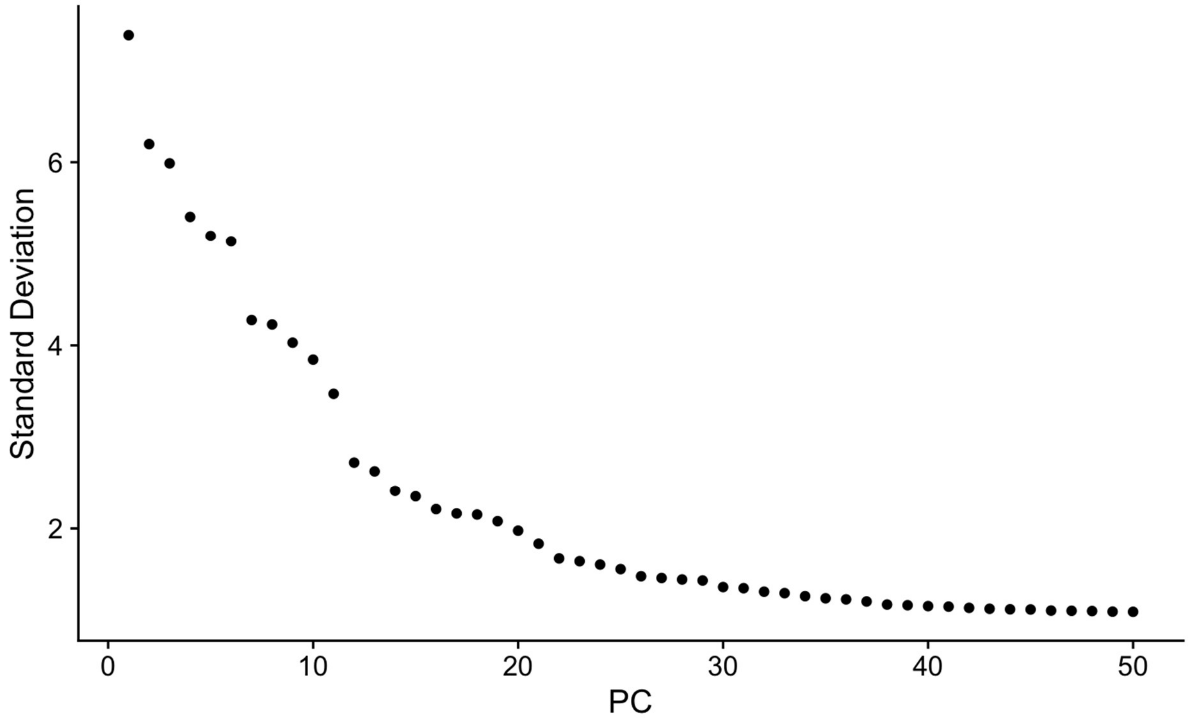

Supplementary Figure 2.

Elbow plot showing a ranking of principal components based upon the percentage of variance explained by each. Here we observe an ‘elbow’ around principal components 30-40, suggesting that the majority of true signal is captured in the first 40 principal components.

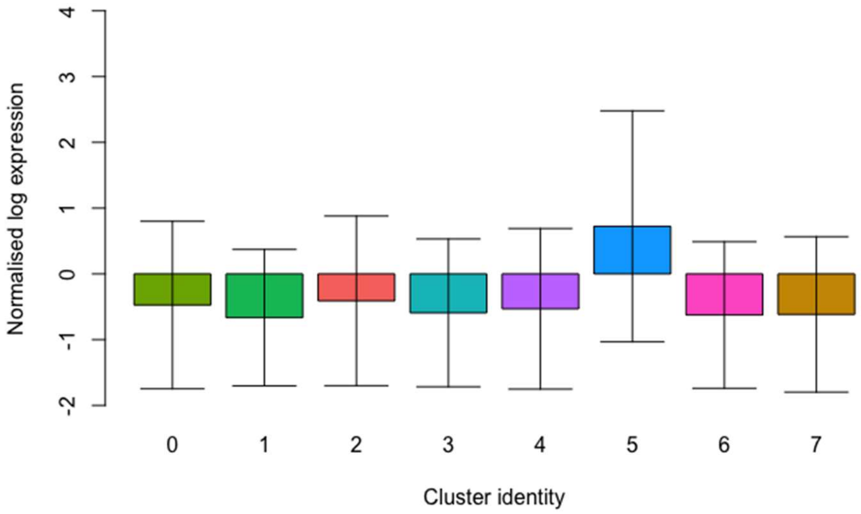

Supplementary Figure 3.

A bar chart showing the normalised log-transformed expression of immunoglobulin genes in each of the B cell sub-clusters. The error bars show the standard deviation.

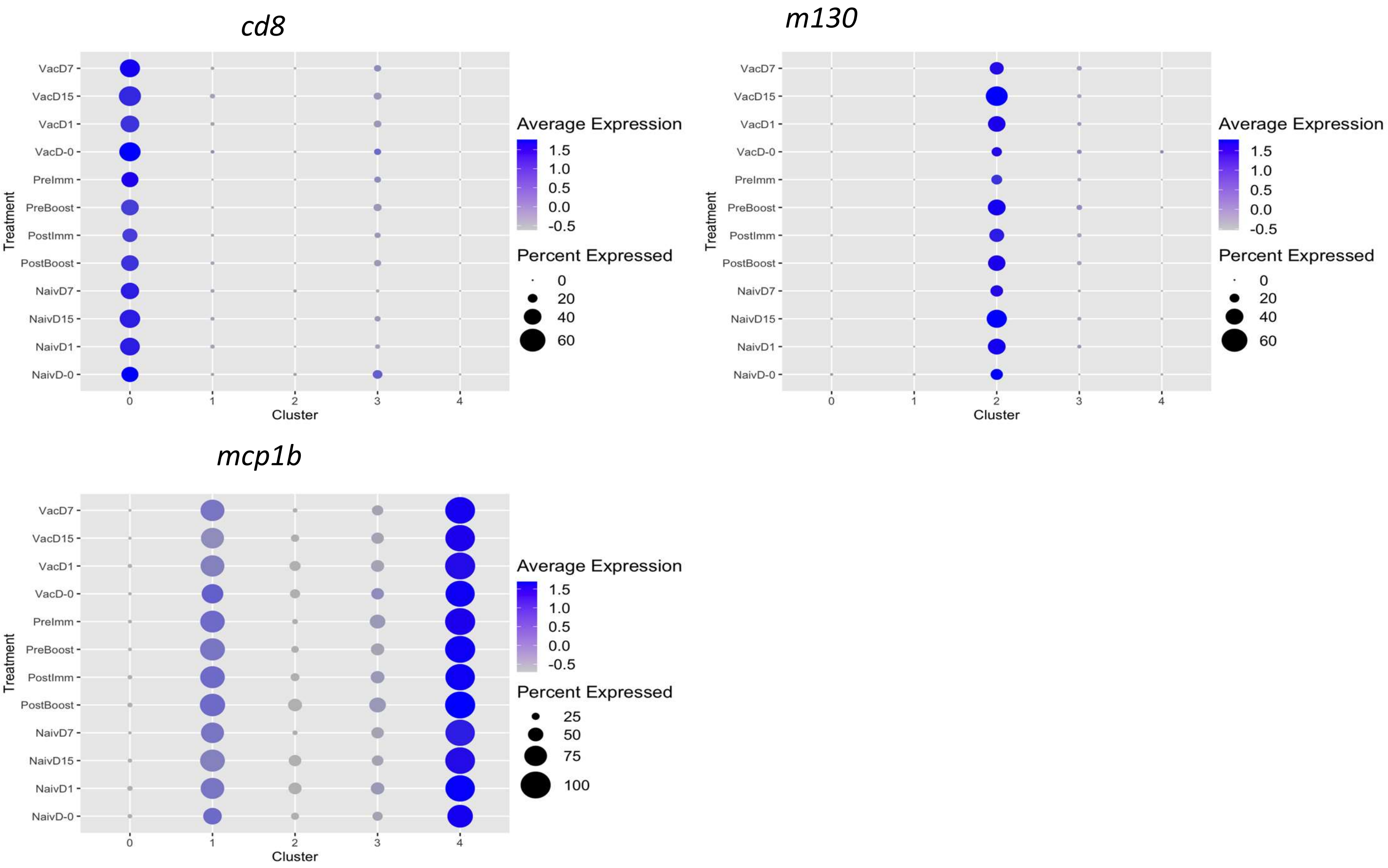

Supplementary Figure 4

Dot plot showing expression of *cd8*, *mcp1b* and *m130* genes in T-cell subclusters across vaccination and immune challenge study timeline. The size of the dot encodes the percentage of cells within a subcluster expressing the gene, while the colour intensity encodes the average expression level of 'expressing' cells.

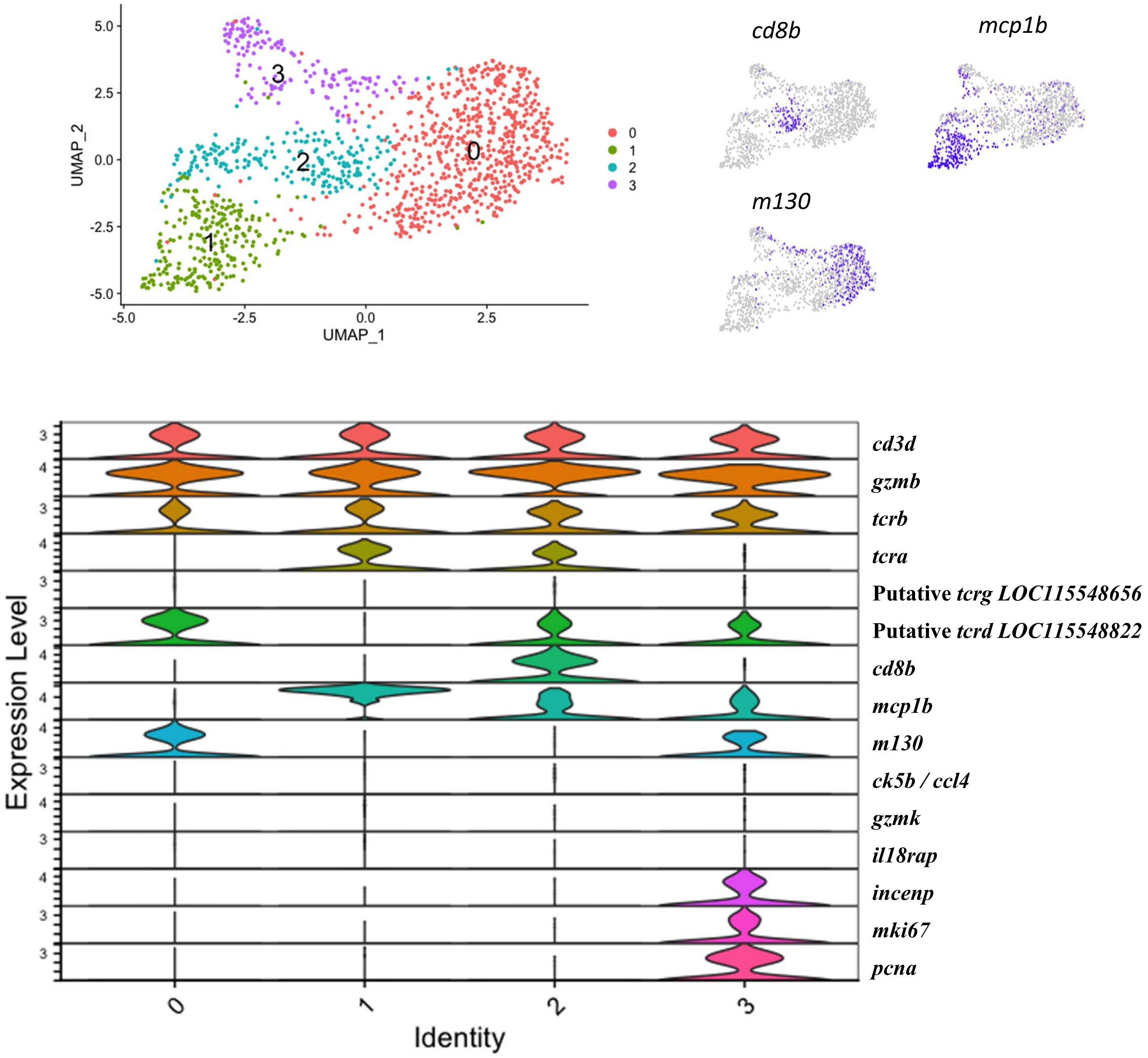

Supplementary figure 5.

Atlantic cod T lymphocytes sub-clusters from previous work. (Guslund et al., 2020)
A) 1,304 T lymphocyte cells clustered at a resolution of 0.3 using UMAP reveals 4 sub-clusters.
B) Violin plots showing expression of selected genes identified by differential expressed gene analysis. The *y-axis* indicates normalized and log-transformed average expression of the selected genes. The *x-axis* indicates the T cell cluster identity shown in the UMAP.
C) Feature plot showing the expression of T cell co-receptor *cd8b*, cytokine *mcp1b* and *m130*.

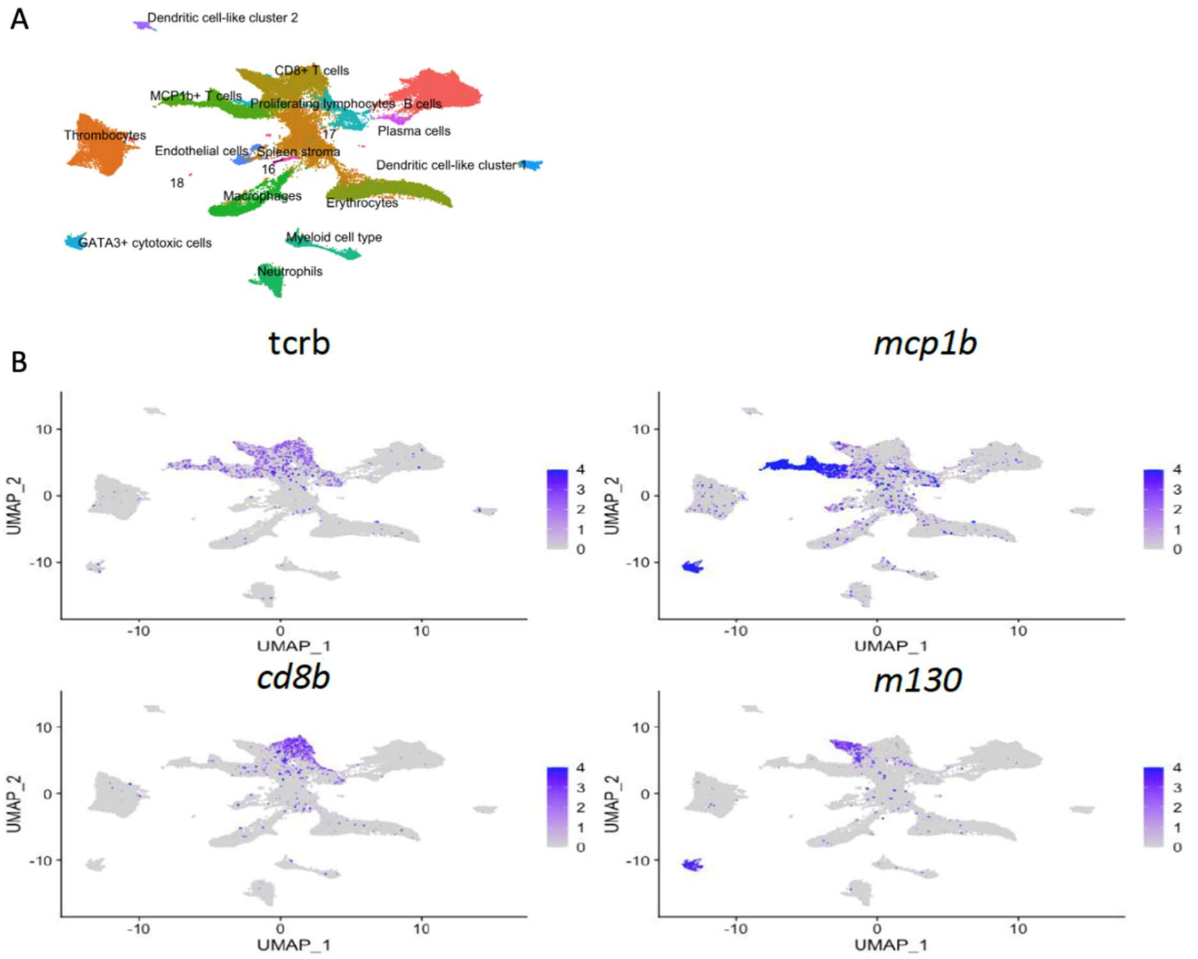

Supplementary Figure 6

Figure showing the expression of key T cell marker genes. (A) Global UMAP containing all splenic cells showing identity of cell clusters. **(B)** Feature plots showing the expression of the T cell receptor tcrb, T cell co-receptor *cd8b*, cytokine *mcp1b*, and *m130*, on the UMAP from the sampled Atlantic cod.

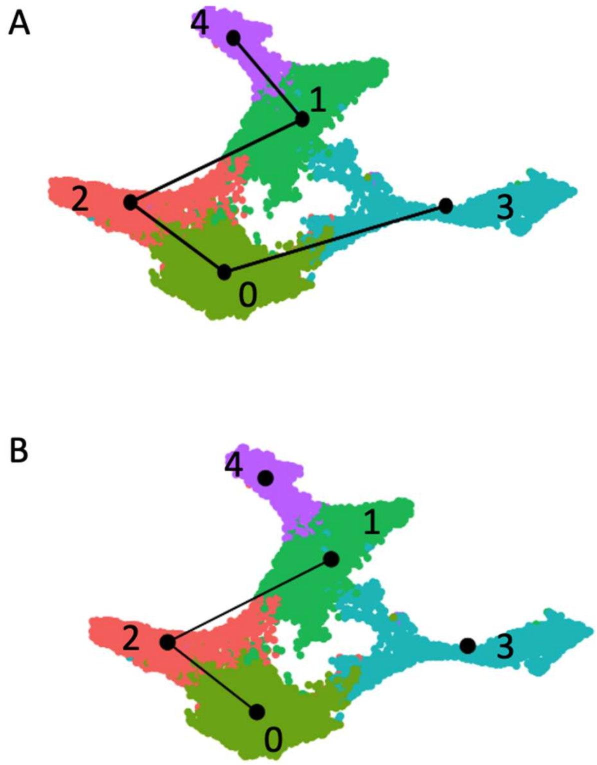

Supplementary Figure 7

Pseudotime analysis of T lymphocytes using Slingshot. **(A)** T cell sub-clusters depicting one Slingshot-predicted lineage trajectory, starting at lineage 3 and ending at lineage 4. **(B)** Selecting multiple trajectories suggests sub-clusters 3 and 4 are separate lineages, while sub-clusters 0, 1 and 3 are connected.

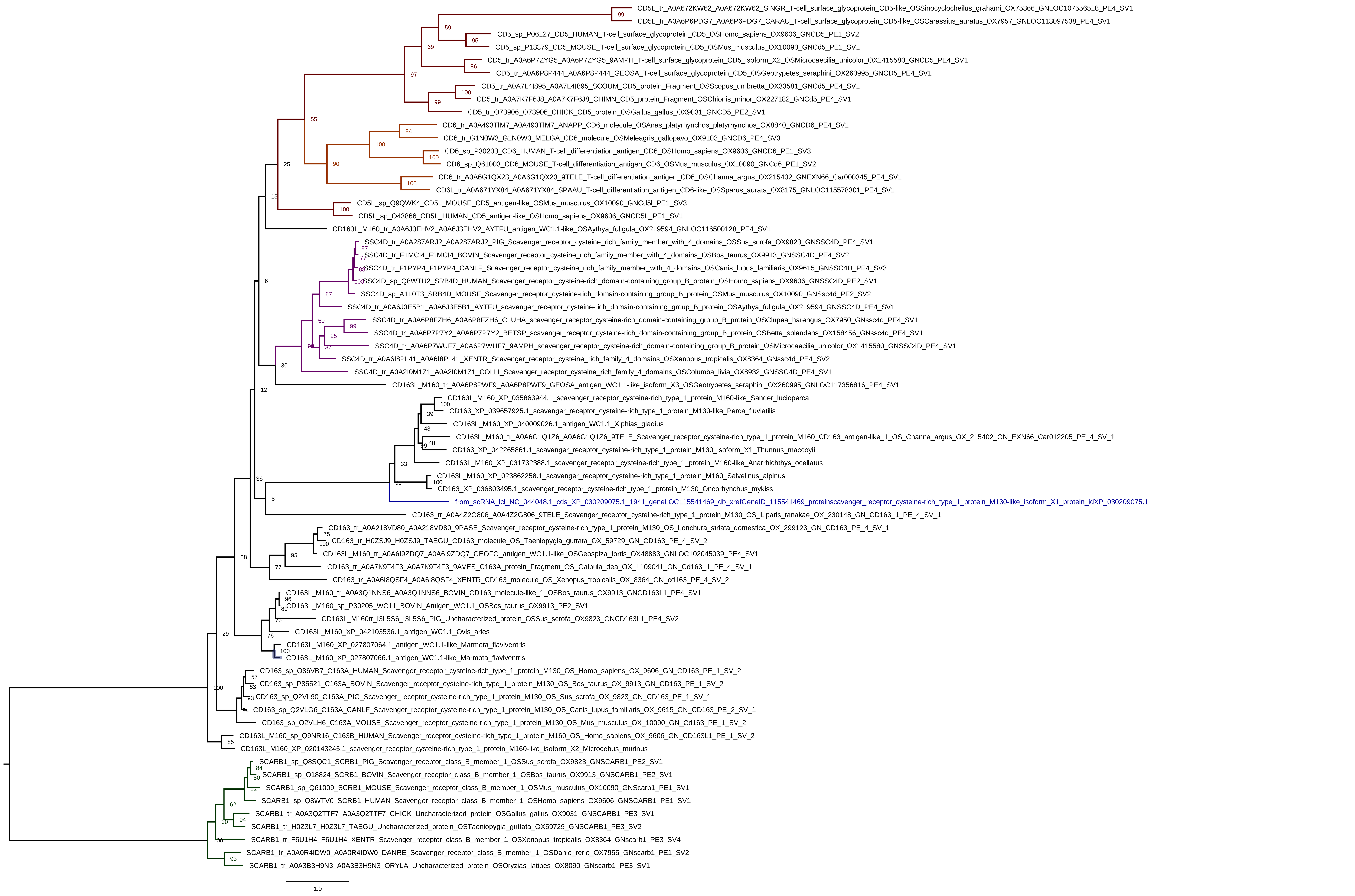
CD5L_tr_A0A672KW62_A0A672KW62_SINGR_T-cell_surface_glycoprotein_CD5-like_OSSinocyclocheilus_grahami_OX75366_GNLOC107556518_PE4_SV1
CD5L_tr_A0A6P6PDG7_A0A6P6PDG7_CARAU_T-cell_surface_glycoprotein_CD5-like_OSCarassius_auratus_OX7957_GNLOC113097538_PE4_SV1
CD5_sp_P06127_CD5_HUMAN_T-cell_surface_glycoprotein_CD5_OSHomo_sapiens_OX9606_GNCD5_PE1_SV2
CD5_sp_P13379_CD5_MOUSE_T-cell_surface_glycoprotein_CD5_OSMus_musculus_OX10090_GNCd5_PE1_SV1
CD5_tr_A0A6P7ZYG5_A0A6P7ZYG5_9AMPH_T-cell_surface_glycoprotein_CD5_isoform_X2_OSMicrocaecilia_unicolor_OX1415580_GNCD5_PE4_SV1
CD5_tr_A0A6P8P444_A0A6P8P444_GEOSA_T-cell_surface_glycoprotein_CD5_OSGeotrypetes_seraphini_OX260995_GNCD5_PE4_SV1
CD5_tr_A0A7L4I895_A0A7L4I895_SCOUM_CD5_protein_Fragment_OSScopus_umbretta_OX33581_GNCd5_PE4_SV1
CD5_tr_A0A7K7F6J8_A0A7K7F6J8_CHIMN_CD5_protein_Fragment_OSChionis_minor_OX227182_GNCd5_PE4_SV1
CD5_tr_O73906_O73906_CHICK_CD5_protein_OSGallus_gallus_OX9031_GNCD5_PE2_SV1
CD6_tr_A0A493TIM7_A0A493TIM7_ANAPP_CD6_molecule_OSAnas_platyrhynchos_platyrhynchos_OX8840_GNCD6_PE4_SV1
CD6_tr_G1N0W3_G1N0W3_MELGA_CD6_molecule_OSMeleagris_gallopavo_OX9103_GNCD6_PE4_SV3
CD6_sp_P30203_CD6_HUMAN_T-cell_differentiation_antigen_CD6_OSHomo_sapiens_OX9606_GNCD6_PE1_SV3
CD6_sp_Q61003_CD6_MOUSE_T-cell_differentiation_antigen_CD6_OSMus_musculus_OX10090_GNCd6_PE1_SV2
CD6_tr_A0A6G1QX23_A0A6G1QX23_9TELE_T-cell_differentiation_antigen_CD6_OSChanna_argus_OX215402_GNEXN66_Car000345_PE4_SV1
CD6L_tr_A0A671YX84_A0A671YX84_SPAAU_T-cell_differentiation_antigen_CD6-like_OSSparus_aurata_OX8175_GNLOC115578301_PE4_SV1
CD5L_sp_Q9QWK4_CD5L_MOUSE_CD5_antigen-like_OSMus_musculus_OX10090_GNCd5l_PE1_SV3
CD5L_sp_O43866_CD5L_HUMAN_CD5_antigen-like_OSHomo_sapiens_OX9606_GNCD5L_PE1_SV1
CD163L_M160_tr_A0A6J3EHV2_A0A6J3EHV2_AYTFU_antigen_WC1.1-like_OSAythya_fuligula_OX219594_GNLOC116500128_PE4_SV1
SSC4D_tr_A0A287ARJ2_A0A287ARJ2_PIG_Scavenger_receptor_cysteine_rich_family_member_with_4_domains_OSSus_scrofa_OX9823_GNSSC4D_PE4_SV1
SSC4D_tr_F1MCI4_F1MCI4_BOVIN_Scavenger_receptor_cysteine_rich_family_member_with_4_domains_OSBos_taurus_OX9913_GNSSC4D_PE4_SV2
SSC4D_tr_F1PYP4_F1PYP4_CANLF_Scavenger_receptor_cysteine_rich_family_member_with_4_domains_OSCanis_lupus_familiaris_OX9615_GNSSC4D_PE4_SV3
SSC4D_sp_Q8WTU2_SRB4D_HUMAN_Scavenger_receptor_cysteine-rich_domain-containing_group_B_protein_OSHomo_sapiens_OX9606_GNSSC4D_PE2_SV1
SSC4D_sp_A1L0T3_SRB4D_MOUSE_Scavenger_receptor_cysteine-rich_domain-containing_group_B_protein_OSMus_musculus_OX10090_GNSsc4d_PE2_SV2
SSC4D_tr_A0A6J3E5B1_A0A6J3E5B1_AYTFU_scavenger_receptor_cysteine-rich_domain-containing_group_B_protein_OSAythya_fuligula_OX219594_GNSSC4D_PE4_SV1
SSC4D_tr_A0A6P8FZH6_A0A6P8FZH6_CLUHA_scavenger_receptor_cysteine-rich_domain-containing_group_B_protein_OSClupea_harengus_OX7950_GNssc4d_PE4_SV1
SSC4D_tr_A0A6P7P7Y2_A0A6P7P7Y2_BETSP_scavenger_receptor_cysteine-rich_domain-containing_group_B_protein_OSBetta_splendens_OX158456_GNssc4d_PE4_SV1
SSC4D_tr_A0A6P7WUF7_A0A6P7WUF7_9AMPH_scavenger_receptor_cysteine-rich_domain-containing_group_B_protein_OSMicrocaecilia_unicolor_OX1415580_GNSSC4D_PE4_SV1
SSC4D_tr_A0A6I8PL41_A0A6I8PL41_XENTR_Scavenger_receptor_cysteine_rich_family_4_domains_OSXenopus_tropicalis_OX8364_GNssc4d_PE4_SV2
SSC4D_tr_A0A2I0M1Z1_A0A2I0M1Z1_COLLI_Scavenger_receptor_cysteine_rich_family_4_domains_OSColumba_livia_OX8932_GNSSC4D_PE4_SV1
CD163L_M160_tr_A0A6P8PWF9_A0A6P8PWF9_GEOSA_antigen_WC1.1-like_isoform_X3_OSGeotrypetes_seraphini_OX260995_GNLOC117356816_PE4_SV1
CD163L_M160_XP_035863944.1_scavenger_receptor_cysteine-rich_type_1_protein_M160-like_Sander_lucioperca
CD163_XP_039657925.1_scavenger_receptor_cysteine-rich_type_1_protein_M130-like_Perca_fluviatilis
CD163L_M160_XP_040009026.1_antigen_WC1.1_Xiphias_gladius
CD163L_M160_tr_A0A6G1Q1Z6_A0A6G1Q1Z6_9TELE_Scavenger_receptor_cysteine-rich_type_1_protein_M160_CD163_antigen-like_1_OS_Channa_argus_OX_215402_GN_EXN66_Car012205_PE_4_SV_1
CD163_XP_042265861.1_scavenger_receptor_cysteine-rich_type_1_protein_M130_isoform_X1_Thunnus_maccoyii
CD163L_M160_XP_031732388.1_scavenger_receptor_cysteine-rich_type_1_protein_M160-like_Anarrhichthys_ocellatus
CD163L_M160_XP_023862258.1_scavenger_receptor_cysteine-rich_type_1_protein_M160_Salvelinus_alpinus
CD163_XP_036803495.1_scavenger_receptor_cysteine-rich_type_1_protein_M130_Oncorhynchus_mykiss
from_scRNA_lcl_NC_044048.1_cds_XP_030209075.1_1941_geneLOC115541469_db_xrefGeneID_115541469_proteinscavenger_receptor_cysteine-rich_type_1_protein_M130-like_isoform_X1_protein_idXP_030209075.1
CD163_tr_A0A4Z2G806_A0A4Z2G806_9TELE_Scavenger_receptor_cysteine-rich_type_1_protein_M130_OS_Liparis_tanakae_OX_230148_GN_CD163_1_PE_4_SV_1
CD163_tr_A0A218VD80_A0A218VD80_9PASE_Scavenger_receptor_cysteine-rich_type_1_protein_M130_OS_Lonchura_striata_domestica_OX_299123_GN_CD163_PE_4_SV_1
CD163_tr_H0ZSJ9_H0ZSJ9_TAEGU_CD163_molecule_OS_Taeniopygia_guttata_OX_59729_GN_CD163_PE_4_SV_2
CD163L_M160_tr_A0A6I9ZDQ7_A0A6I9ZDQ7_GEOFO_antigen_WC1.1-like_OSGeospiza_fortis_OX48883_GNLOC102045039_PE4_SV1
CD163_tr_A0A7K9T4F3_A0A7K9T4F3_9AVES_C163A_protein_Fragment_OS_Galbula_dea_OX_1109041_GN_Cd163_1_PE_4_SV_1
CD163_tr_A0A6I8QSF4_A0A6I8QSF4_XENTR_CD163_molecule_OS_Xenopus_tropicalis_OX_8364_GN_cd163_PE_4_SV_2
CD163L_M160_tr_A0A3Q1NNS6_A0A3Q1NNS6_BOVIN_CD163_molecule-like_1_OSBos_taurus_OX9913_GNCD163L1_PE4_SV1
CD163L_M160_sp_P30205_WC11_BOVIN_Antigen_WC1.1_OSBos_taurus_OX9913_PE2_SV1
CD163L_M160tr_I3L5S6_I3L5S6_PIG_Uncharacterized_protein_OSSus_scrofa_OX9823_GNCD163L1_PE4_SV2
CD163L_M160_XP_042103536.1_antigen_WC1.1_Ovis_aries
CD163L_M160_XP_027807064.1_antigen_WC1.1-like_Marmota_flaviventris
CD163L_M160_XP_027807066.1_antigen_WC1.1-like_Marmota_flaviventris
CD163_sp_Q86VB7_C163A_HUMAN_Scavenger_receptor_cysteine-rich_type_1_protein_M130_OS_Homo_sapiens_OX_9606_GN_CD163_PE_1_SV_2
CD163_sp_P85521_C163A_BOVIN_Scavenger_receptor_cysteine-rich_type_1_protein_M130_OS_Bos_taurus_OX_9913_GN_CD163_PE_1_SV_2
CD163_sp_Q2VL90_C163A_PIG_Scavenger_receptor_cysteine-rich_type_1_protein_M130_OS_Sus_scrofa_OX_9823_GN_CD163_PE_1_SV_1
CD163_sp_Q2VLG6_C163A_CANLF_Scavenger_receptor_cysteine-rich_type_1_protein_M130_OS_Canis_lupus_familiaris_OX_9615_GN_CD163_PE_2_SV_1
CD163_sp_Q2VLH6_C163A_MOUSE_Scavenger_receptor_cysteine-rich_type_1_protein_M130_OS_Mus_musculus_OX_10090_GN_Cd163_PE_1_SV_2
CD163L_M160_sp_Q9NR16_C163B_HUMAN_Scavenger_receptor_cysteine-rich_type_1_protein_M160_OS_Homo_sapiens_OX_9606_GN_CD163L1_PE_1_SV_2
CD163L_M160_XP_020143245.1_scavenger_receptor_cysteine-rich_type_1_protein_M160-like_isoform_X2_Microcebus_murinus
SCARB1_sp_Q8SQC1_SCRB1_PIG_Scavenger_receptor_class_B_member_1_OSSus_scrofa_OX9823_GNSCARB1_PE2_SV1
SCARB1_sp_O18824_SCRB1_BOVIN_Scavenger_receptor_class_B_member_1_OSBos_taurus_OX9913_GNSCARB1_PE2_SV1
SCARB1_sp_Q61009_SCRB1_MOUSE_Scavenger_receptor_class_B_member_1_OSMus_musculus_OX10090_GNScarb1_PE1_SV1
SCARB1_sp_Q8WTV0_SCRB1_HUMAN_Scavenger_receptor_class_B_member_1_OSHomo_sapiens_OX9606_GNSCARB1_PE1_SV1
SCARB1_tr_A0A3Q2TTF7_A0A3Q2TTF7_CHICK_Uncharacterized_protein_OSGallus_gallus_OX9031_GNSCARB1_PE3_SV1
SCARB1_tr_H0Z3L7_H0Z3L7_TAEGU_Uncharacterized_protein_OSTaeniopygia_guttata_OX59729_GNSCARB1_PE3_SV2
SCARB1_tr_F6U1H4_F6U1H4_XENTR_Scavenger_receptor_class_B_member_1_OSXenopus_tropicalis_OX8364_GNscarb1_PE3_SV4
SCARB1_tr_A0A0R4IDW0_A0A0R4IDW0_DANRE_Scavenger_receptor_class_B_member_1_OSDanio_rerio_OX7955_GNscarb1_PE1_SV2
SCARB1_tr_A0A3B3H9N3_A0A3B3H9N3_ORYLA_Uncharacterized_protein_OSOryzias_latipes_OX8090_GNscarb1_PE3_SV1
1.0

Supplementary Figure 8A

Neighbour joining phylogram of CD163/Scavenger receptor M160/M130 from Atlantic cod (Gadus morhua) and selected other species. Scavenger Receptor Cysteine-Rich Type 1 Protein M130 (CD163) references were collected from the groups Mammalia, Aves, Reptilia, Amphibia and Teleostei at Uniprot.org where available. The predicted gene models of the scRNA-reported annotations were then subjected to a blastp at NCBI towards mammalian and teleostei databases using default parameters. Here, hits annotated as M130/M160/CD163 were randomly selected to be included in the multiple sequence alignment. Members of the group B SRCR family were included (CD5, CD5L and CD6) together with two other scavenger receptors: scavenger receptor cysteine rich family member with 4 domains (SSC4D) and Scavenger Receptor Class B Member 1 (SCARB1). A multiple sequence alignment was generated using MEGA7 and its MUSCLE alignment program using default setting. A neighbour joining tree using Poisson distribution, pairwise deletion and 300 bootstrap replicates was made using MEGA7. The resulting tree was imported into FigTree for annotations and presentation. Root placed on the SCARB1 clade is coloured green. Members of the superfamily beyond CD163 and CD163L colored magenta (SSC4D), red (CD5 and CD5L) and orange (CD6). The *mcb1b* gene model from scRNA analyses is coloured blue.

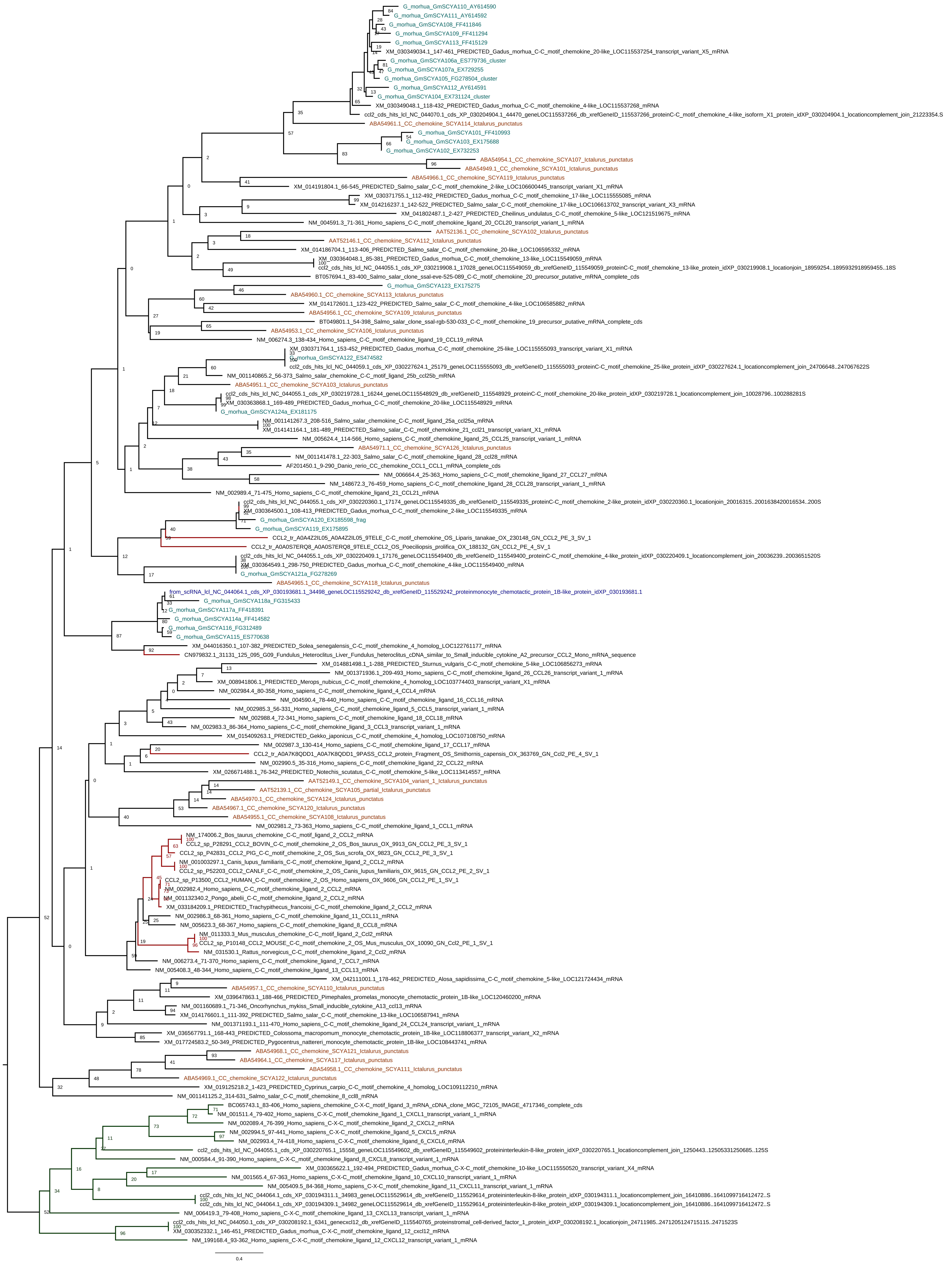

G_morhua_GmSCYA110_AY614590
G_morhua_GmSCYA111_AY614592
G_morhua_GmSCYA108_FF411846
G_morhua_GmSCYA109_FF411294
G_morhua_GmSCYA113_FF415129
XM_030349034.1_147-461_PREDICTED_Gadus_morhua_C-C_motif_chemokine_20-like_LOC115537254_transcript_variant_X5_mRNA
G_morhua_GmSCYA106a_ES779736_cluster
G_morhua_GmSCYA107a_EX729255
G_morhua_GmSCYA105_FG278504_cluster
G_morhua_GmSCYA112_AY614591
G_morhua_GmSCYA104_EX731124_cluster
XM_030349048.1_118-432_PREDICTED_Gadus_morhua_C-C_motif_chemokine_4-like_LOC115537268_mRNA
ccl2_cds_hits_lcl_NC_044070.1_cds_XP_030204904.1_44470_geneLOC115537266_db_xrefGeneID_115537266_proteinC-C_motif_chemokine_4-like_isoform_X1_protein_idXP_030204904.1_locationcomplement_join_21223354.S
ABA54961.1_CC_chemokine_SCYA114_Ictalurus_punctatus
G_morhua_GmSCYA101_FF410993
G_morhua_GmSCYA103_EX175688
G_morhua_GmSCYA102_EX732253
ABA54954.1_CC_chemokine_SCYA107_Ictalurus_punctatus
ABA54949.1_CC_chemokine_SCYA101_Ictalurus_punctatus
ABA54966.1_CC_chemokine_SCYA119_Ictalurus_punctatus
XM_014191804.1_66-545_PREDICTED_Salmo_salar_C-C_motif_chemokine_2-like_LOC106600445_transcript_variant_X1_mRNA
XM_030371755.1_112-492_PREDICTED_Gadus_morhua_C-C_motif_chemokine_17-like_LOC115555085_mRNA
XM_014216237.1_142-522_PREDICTED_Salmo_salar_C-C_motif_chemokine_17-like_LOC106613702_transcript_variant_X3_mRNA
XM_041802487.1_2-427_PREDICTED_Cheilinus_undulatus_C-C_motif_chemokine_5-like_LOC121519675_mRNA
NM_004591.3_71-361_Homo_sapiens_C-C_motif_chemokine_ligand_20_CCL20_transcript_variant_1_mRNA
AAT52136.1_CC_chemokine_SCYA102_Ictalurus_punctatus
AAT52146.1_CC_chemokine_SCYA112_Ictalurus_punctatus
XM_014186704.1_113-406_PREDICTED_Salmo_salar_C-C_motif_chemokine_20-like_LOC106595332_mRNA
XM_030364048.1_85-381_PREDICTED_Gadus_morhua_C-C_motif_chemokine_13-like_LOC115549059_mRNA
ccl2_cds_hits_lcl_NC_044055.1_cds_XP_030219908.1_17028_geneLOC115549059_db_xrefGeneID_115549059_proteinC-C_motif_chemokine_13-like_protein_idXP_030219908.1_locationjoin_18959254..1895932918959455..18S
BT057694.1_83-400_Salmo_salar_clone_ssal-eve-525-089_C-C_motif_chemokine_20_precursor_putative_mRNA_complete_cds
G_morhua_GmSCYA123_EX175275
ABA54960.1_CC_chemokine_SCYA113_Ictalurus_punctatus
XM_014172601.1_123-422_PREDICTED_Salmo_salar_C-C_motif_chemokine_4-like_LOC106585882_mRNA
ABA54956.1_CC_chemokine_SCYA109_Ictalurus_punctatus
BT049801.1_54-398_Salmo_salar_clone_ssal-rgb-530-033_C-C_motif_chemokine_19_precursor_putative_mRNA_complete_cds
ABA54953.1_CC_chemokine_SCYA106_Ictalurus_punctatus
NM_006274.3_138-434_Homo_sapiens_C-C_motif_chemokine_ligand_19_CCL19_mRNA
XM_030371764.1_153-452_PREDICTED_Gadus_morhua_C-C_motif_chemokine_25-like_LOC115555093_transcript_variant_X1_mRNA
G_morhua_GmSCYA122_ES474582
ccl2_cds_hits_lcl_NC_044059.1_cds_XP_030227624.1_25179_geneLOC115555093_db_xrefGeneID_115555093_proteinC-C_motif_chemokine_25-like_protein_idXP_030227624.1_locationcomplement_join_24706648..247067622S
NM_001140865.2_56-373_Salmo_salar_chemokine_C-C_motif_ligand_25b_ccl25b_mRNA
ABA54951.1_CC_chemokine_SCYA103_Ictalurus_punctatus
ccl2_cds_hits_lcl_NC_044055.1_cds_XP_030219728.1_16244_geneLOC115548929_db_xrefGeneID_115548929_proteinC-C_motif_chemokine_20-like_protein_idXP_030219728.1_locationcomplement_join_10028796..100288281S
XM_030363868.1_169-489_PREDICTED_Gadus_morhua_C-C_motif_chemokine_20-like_LOC115548929_mRNA
G_morhua_GmSCYA124a_EX181175
NM_001141267.3_208-516_Salmo_salar_chemokine_C-C_motif_ligand_25a_ccl25a_mRNA
XM_014141164.1_181-489_PREDICTED_Salmo_salar_C-C_motif_chemokine_21_ccl21_transcript_variant_X1_mRNA
NM_005624.4_114-566_Homo_sapiens_C-C_motif_chemokine_ligand_25_CCL25_transcript_variant_1_mRNA
ABA54971.1_CC_chemokine_SCYA126_Ictalurus_punctatus
NM_001141478.1_22-303_Salmo_salar_C-C_motif_chemokine_ligand_28_ccl28_mRNA
AF201450.1_9-290_Danio_rerio_CC_chemokine_CCL1_CCL1_mRNA_complete_cds
NM_006664.4_25-363_Homo_sapiens_C-C_motif_chemokine_ligand_27_CCL27_mRNA
NM_148672.3_76-459_Homo_sapiens_C-C_motif_chemokine_ligand_28_CCL28_transcript_variant_1_mRNA
NM_002989.4_71-475_Homo_sapiens_C-C_motif_chemokine_ligand_21_CCL21_mRNA
ccl2_cds_hits_lcl_NC_044055.1_cds_XP_030220360.1_17174_geneLOC115549335_db_xrefGeneID_115549335_proteinC-C_motif_chemokine_2-like_protein_idXP_030220360.1_locationjoin_20016315..2001638420016534..200S
XM_030364500.1_108-413_PREDICTED_Gadus_morhua_C-C_motif_chemokine_2-like_LOC115549335_mRNA
G_morhua_GmSCYA120_EX185598_frag
G_morhua_GmSCYA119_EX175895
CCL2_tr_A0A4Z2IL05_A0A4Z2IL05_9TELE_C-C_motif_chemokine_OS_Liparis_tanakae_OX_230148_GN_CCL2_PE_3_SV_1
CCL2_tr_A0A0S7ERQ8_A0A0S7ERQ8_9TELE_CCL2_OS_Poeciliopsis_prolifica_OX_188132_GN_CCL2_PE_4_SV_1
ccl2_cds_hits_lcl_NC_044055.1_cds_XP_030220409.1_17176_geneLOC115549400_db_xrefGeneID_115549400_proteinC-C_motif_chemokine_4-like_protein_idXP_030220409.1_locationcomplement_join_20036239..2003651520S
XM_030364549.1_298-750_PREDICTED_Gadus_morhua_C-C_motif_chemokine_4-like_LOC115549400_mRNA
G_morhua_GmSCYA121a_FG278269
ABA54965.1_CC_chemokine_SCYA118_Ictalurus_punctatus
from_scRNA_lcl_NC_044064.1_cds_XP_030193681.1_34498_geneLOC115529242_db_xrefGeneID_115529242_proteinmonocyte_chemotactic_protein_1B-like_protein_idXP_030193681.1
G_morhua_GmSCYA118a_FG315433
G_morhua_GmSCYA117a_FF418391
G_morhua_GmSCYA114a_FF414582
G_morhua_GmSCYA116_FG312489
G_morhua_GmSCYA115_ES770638
XM_044016350.1_107-382_PREDICTED_Solea_senegalensis_C-C_motif_chemokine_4_homolog_LOC122761177_mRNA
CN979832.1_31131_125_095_G09_Fundulus_Heteroclitus_Liver_Fundulus_heteroclitus_cDNA_similar_to_Small_inducible_cytokine_A2_precursor_CCL2_Mono_mRNA_sequence
XM_014881498.1_1-288_PREDICTED_Sturnus_vulgaris_C-C_motif_chemokine_5-like_LOC106856273_mRNA
NM_001371936.1_209-493_Homo_sapiens_C-C_motif_chemokine_ligand_26_CCL26_transcript_variant_1_mRNA
XM_008941806.1_PREDICTED_Merops_nubicus_C-C_motif_chemokine_4_homolog_LOC103774403_transcript_variant_X1_mRNA
NM_002984.4_80-358_Homo_sapiens_C-C_motif_chemokine_ligand_4_CCL4_mRNA
NM_004590.4_78-440_Homo_sapiens_C-C_motif_chemokine_ligand_16_CCL16_mRNA
NM_002985.3_56-331_Homo_sapiens_C-C_motif_chemokine_ligand_5_CCL5_transcript_variant_1_mRNA
NM_002988.4_72-341_Homo_sapiens_C-C_motif_chemokine_ligand_18_CCL18_mRNA
NM_002983.3_86-364_Homo_sapiens_C-C_motif_chemokine_ligand_3_CCL3_transcript_variant_1_mRNA
XM_015409263.1_PREDICTED_Gekko_japonicus_C-C_motif_chemokine_4_homolog_LOC107108750_mRNA
NM_002987.3_130-414_Homo_sapiens_C-C_motif_chemokine_ligand_17_CCL17_mRNA
CCL2_tr_A0A7K8QDD1_A0A7K8QDD1_9PASS_CCL2_protein_Fragment_OS_Smithornis_capensis_OX_363769_GN_Ccl2_PE_4_SV_1
NM_002990.5_35-316_Homo_sapiens_C-C_motif_chemokine_ligand_22_CCL22_mRNA
XM_026671488.1_76-342_PREDICTED_Notechis_scutatus_C-C_motif_chemokine_5-like_LOC113414557_mRNA
AAT52149.1_CC_chemokine_SCYA104_variant_1_Ictalurus_punctatus
AAT52139.1_CC_chemokine_SCYA105_partial_Ictalurus_punctatus
ABA54970.1_CC_chemokine_SCYA124_Ictalurus_punctatus
ABA54967.1_CC_chemokine_SCYA120_Ictalurus_punctatus
ABA54955.1_CC_chemokine_SCYA108_Ictalurus_punctatus
NM_002981.2_73-363_Homo_sapiens_C-C_motif_chemokine_ligand_1_CCL1_mRNA
NM_174006.2_Bos_taurus_chemokine_C-C_motif_ligand_2_CCL2_mRNA
CCL2_sp_P28291_CCL2_BOVIN_C-C_motif_chemokine_2_OS_Bos_taurus_OX_9913_GN_CCL2_PE_3_SV_1
CCL2_sp_P42831_CCL2_PIG_C-C_motif_chemokine_2_OS_Sus_scrofa_OX_9823_GN_CCL2_PE_3_SV_1
NM_001003297.1_Canis_lupus_familiaris_C-C_motif_chemokine_ligand_2_CCL2_mRNA
CCL2_sp_P52203_CCL2_CANLF_C-C_motif_chemokine_2_OS_Canis_lupus_familiaris_OX_9615_GN_CCL2_PE_2_SV_1
CCL2_sp_P13500_CCL2_HUMAN_C-C_motif_chemokine_2_OS_Homo_sapiens_OX_9606_GN_CCL2_PE_1_SV_1
NM_002982.4_Homo_sapiens_C-C_motif_chemokine_ligand_2_CCL2_mRNA
NM_001132340.2_Pongo_abelii_C-C_motif_chemokine_ligand_2_CCL2_mRNA
XM_033184209.1_PREDICTED_Trachypithecus_francoisi_C-C_motif_chemokine_ligand_2_CCL2_mRNA
NM_002986.3_68-361_Homo_sapiens_C-C_motif_chemokine_ligand_11_CCL11_mRNA
NM_005623.3_68-367_Homo_sapiens_C-C_motif_chemokine_ligand_8_CCL8_mRNA
NM_011333.3_Mus_musculus_chemokine_C-C_motif_ligand_2_Ccl2_mRNA
CCL2_sp_P10148_CCL2_MOUSE_C-C_motif_chemokine_2_OS_Mus_musculus_OX_10090_GN_Ccl2_PE_1_SV_1
NM_031530.1_Rattus_norvegicus_C-C_motif_chemokine_ligand_2_Ccl2_mRNA
NM_006273.4_71-370_Homo_sapiens_C-C_motif_chemokine_ligand_7_CCL7_mRNA
NM_005408.3_48-344_Homo_sapiens_C-C_motif_chemokine_ligand_13_CCL13_mRNA
XM_042111001.1_178-462_PREDICTED_Alosa_sapidissima_C-C_motif_chemokine_5-like_LOC121724434_mRNA
ABA54957.1_CC_chemokine_SCYA110_Ictalurus_punctatus
XM_039647863.1_188-466_PREDICTED_Pimephales_promelas_monocyte_chemotactic_protein_1B-like_LOC120460200_mRNA
NM_001160689.1_71-346_Oncorhynchus_mykiss_Small_inducible_cytokine_A13_ccl13_mRNA
XM_014176601.1_111-392_PREDICTED_Salmo_salar_C-C_motif_chemokine_13-like_LOC106587941_mRNA
NM_001371193.1_111-470_Homo_sapiens_C-C_motif_chemokine_ligand_24_CCL24_transcript_variant_1_mRNA
XM_036567791.1_168-443_PREDICTED_Colossoma_macropomum_monocyte_chemotactic_protein_1B-like_LOC118806377_transcript_variant_X2_mRNA
XM_017724583.2_50-349_PREDICTED_Pygocentrus_nattereri_monocyte_chemotactic_protein_1B-like_LOC108443741_mRNA
ABA54968.1_CC_chemokine_SCYA121_Ictalurus_punctatus
ABA54964.1_CC_chemokine_SCYA117_Ictalurus_punctatus
ABA54958.1_CC_chemokine_SCYA111_Ictalurus_punctatus
ABA54969.1_CC_chemokine_SCYA122_Ictalurus_punctatus
XM_019125218.2_1-423_PREDICTED_Cyprinus_carpio_C-C_motif_chemokine_4_homolog_LOC109112210_mRNA
NM_001141125.2_314-631_Salmo_salar_C-C_motif_chemokine_8_ccl8_mRNA
BC065743.1_83-406_Homo_sapiens_chemokine_C-X-C_motif_ligand_3_mRNA_cDNA_clone_MGC_72105_IMAGE_4717346_complete_cds
NM_001511.4_79-402_Homo_sapiens_C-X-C_motif_chemokine_ligand_1_CXCL1_transcript_variant_1_mRNA
NM_002089.4_76-399_Homo_sapiens_C-X-C_motif_chemokine_ligand_2_CXCL2_mRNA
NM_002994.5_97-441_Homo_sapiens_C-X-C_motif_chemokine_ligand_5_CXCL5_mRNA
NM_002993.4_74-418_Homo_sapiens_C-X-C_motif_chemokine_ligand_6_CXCL6_mRNA
ccl2_cds_hits_lcl_NC_044055.1_cds_XP_030220765.1_15558_geneLOC115549602_db_xrefGeneID_115549602_proteininterleukin-8-like_protein_idXP_030220765.1_locationcomplement_join_1250443..12505331250685..125S
NM_000584.4_91-390_Homo_sapiens_C-X-C_motif_chemokine_ligand_8_CXCL8_transcript_variant_1_mRNA
XM_030365622.1_192-494_PREDICTED_Gadus_morhua_C-X-C_motif_chemokine_10-like_LOC115550520_transcript_variant_X4_mRNA
NM_001565.4_67-363_Homo_sapiens_C-X-C_motif_chemokine_ligand_10_CXCL10_transcript_variant_1_mRNA
NM_005409.5_84-368_Homo_sapiens_C-X-C_motif_chemokine_ligand_11_CXCL11_transcript_variant_1_mRNA
ccl2_cds_hits_lcl_NC_044064.1_cds_XP_030194311.1_34983_geneLOC115529614_db_xrefGeneID_115529614_proteininterleukin-8-like_protein_idXP_030194311.1_locationcomplement_join_16410886..1641099716412472..S
ccl2_cds_hits_lcl_NC_044064.1_cds_XP_030194309.1_34982_geneLOC115529614_db_xrefGeneID_115529614_proteininterleukin-8-like_protein_idXP_030194309.1_locationcomplement_join_16410886..1641099716412472..S
NM_006419.3_79-408_Homo_sapiens_C-X-C_motif_chemokine_ligand_13_CXCL13_transcript_variant_1_mRNA
ccl2_cds_hits_lcl_NC_044050.1_cds_XP_030208192.1_6341_genecxcl12_db_xrefGeneID_115540765_proteinstromal_cell-derived_factor_1_protein_idXP_030208192.1_locationjoin_24711985..2471205124715115..2471523S
XM_030352332.1_146-451_PREDICTED_Gadus_morhua_C-X-C_motif_chemokine_ligand_12_cxcl12_mRNA
NM_199168.4_93-362_Homo_sapiens_C-X-C_motif_chemokine_ligand_12_CXCL12_transcript_variant_1_mRNA
0.4

Supplementary Figure 8B

Neighbour joining phylogram of CCL2/MCP1b from Atlantic cod (Gadus morhua), Channel catfish and selected species. Monocyte chemotactic protein 1 (MCP1/CCL2) references were collected from the groups Mammalia, Aves, Reptilia, Amphibia and Teleostei at Uniprot.org where available. The predicted gene models of the scRNA-reported annotations were then subjected to a blastp at NCBI towards mammalian and teleostei databases using default parameters. A set of references for the most common CC chemokines in humans and fish were downloaded from genbank and added to the alignment. Some CXC chemokines were added to function as a collective outgroup. Finally, complete CC chemokine characterizations from Channel catfish and Atlantic cod were added, together with all predicted gene models from gadMor3 reported from a tblastn search using the CCL2 references, default parameters and e-value cutoff at 1e-1. A multiple sequence alignment was generated using MEGA7 and its MUSCLE alignment program using default setting. A neighbour joining tree using Poisson distribution, pairwise deletion and 300 bootstrap replicates was made using MEGA7. The resulting tree was imported into FigTree for annotations and presentation. Root placed on the CXC chemokines clade is coloured green. CC chemokines characterized in Atlantic cod are coloured teal. CC chemokines characterized in Channel catfish are coloured orange. Genes annotated as CCL2 from databases are coloured red. Gene model from scRNA analyses is coloured blue.

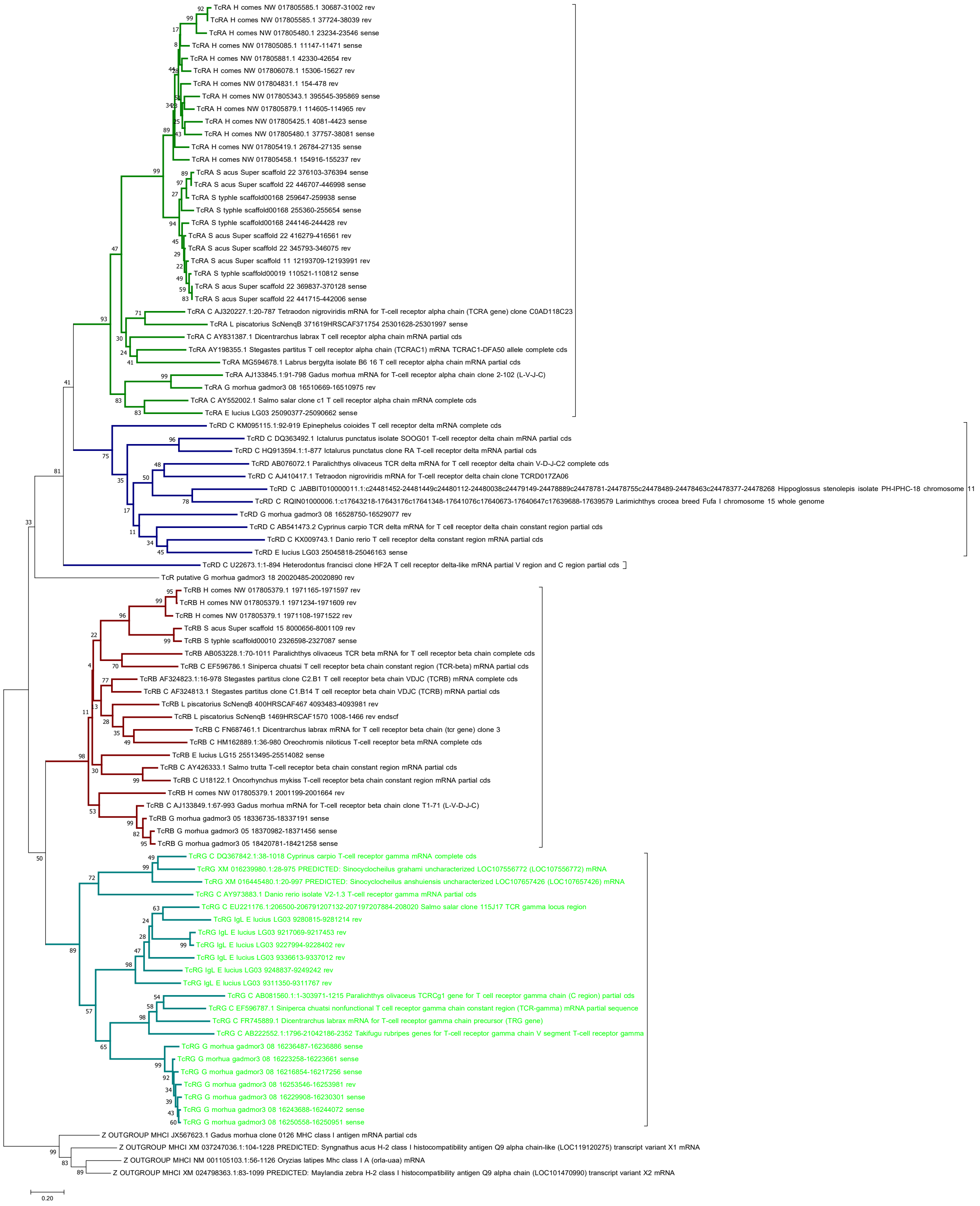

Supplementary Figure 8C

A neighbour joining phylogram of TCR genes in Atlantic cod (Gadus morhua) and selected other species. Annotated *tcrb* (LOC115544273) and *tcra* (LOC115548821) genes in gadMor3, as well as putative TCR genes (LOC115548656 and LOC115548822) identified by blasting gadMor3 with TCR genes from different teleosts, reptiles and birds were compared. All genes with hits above 1e-100 were collected and added to the overall sequence alignment. A multiple sequence alignment was generated using MEGA7 and its MUSCLE alignment program using default setting. From here a neighbor joining tree using Poisson distribution, pairwise deletion and 200 bootstrap replicates was made again using MEGA7. The resulting tree was imported into FigTree for annotations and presentation purposes. LOC115548656 clustered with known tcrg genes and LOC115548822 with known *tcrd* genes suggesting that these two genes represent Atlantic cod *tcrg* and *tcrd*, respectively. Blue text: TCR gene loci mentioned in the manuscript; Teal text: genome constants; Green: TcRA; Mustard: TcRD; Red: TcRB; Blue TcRG
