# Supplementary material for: Lymphocyte subsets in Atlantic cod (Gadus morhua) interrogated by single-cell sequencing
Source: Commun Biol. 2022 Jul 11;5:689. doi: 10.1038/s42003-022-03645-w (PMC9276791; doi:10.1038/s42003-022-03645-w)
Supplement: Supplementary file 3 — Description of Additional Supplementary Files [file 42003_2022_3645_MOESM3_ESM.pdf]

Description of Additional Supplementary Files

File name: Supplementary Data 1
Description: Overview of sequencing, mapping and cells included after filtering.

File name: Supplementary Data 2
Description: Top 20 differentially expressed genes for all splenic cells.

File name: Supplementary Data 3
Description: Top 100 most variable differentially expressed genes across pseudotime for B cells.

File name: Supplementary Data 4
Description: Number of cells present in each named cell cluster.

File name: Supplementary Data 5
Description: B cell sub-clusters top differentially expressed genes.

File name: Supplementary Data 6
Description: T cell sub-clusters top differentially expressed genes.
